# Supplementary material for: A Rapid Review of the Factors That Influence Service User Involvement in Interprofessional Education, Practice, and Research
Source: Int J Environ Res Public Health. 2022 Dec 15;19(24):16826. doi: 10.3390/ijerph192416826 (PMC9779295; doi:10.3390/ijerph192416826)
Supplement: Supplementary file 1 [file ijerph-19-16826-s001.zip › ijerph-2056090-supplementary.pdf]

Supplementary Table S1
WHO minimum reporting items for rapid reviews checklist

Category	Items to consider	Response
Protocol	Was a protocol used?	Yes
	If so, was the protocol made public, published in a journal, and/or registered (if so, provide reference and/or registration number, or link to protocol)?	Yes, it was registered on Open Science Framework – https://osf.io/p5f2w/<br>It was also uploaded on the ResearchGate webpage of all the authors
Overall scope	Was the scope limited in any way?	No
	Were there a limited number of research or policy questions?	No
	Were the research questions of limited type (e.g. effectiveness only, specific populations)?	No
	Was the number of included studies limited?	No
Comprehensiveness	Was the search strategy limited in any way (e.g. number of databases, grey literature, date, setting, language)?	Yes. Databases were limited to Cochrane Central, MEDLINE via Ovid, Embase, and CINAHL via EBSCO. This number well-exceeded the number of databases required to be searched for a rapid review.
	Were there limits on the types of study designs included (e.g. existing systematic reviews, randomized controlled trials)?	No. Quantitative, qualitative, and mixed methods studies were included.
	Was textual analysis limited (e.g. no full-text review and/or limits on the number of items extracted)?	No
Rigour and quality control	Was the process of dual study selection or dual data extraction modified or omitted?	No
	Was the internal or external review of the final research limited or omitted?	Yes
Synthesis	Was the assessment of risk of bias or quality of evidence limited or omitted?	No

	Was qualitative or quantitative analysis limited or omitted?	No
Other	When making statements about the findings of the rapid review, were the conclusions simplified or omitted?	No
	Is it appropriate to provide a disclaimer and/or limitations section in context with your findings?	Yes. Limitations of the review have been outlined.

Supplementary Table S2
Search strings used for each electronic database

Database	Search String
CINAHL via EBSCO	(((MH "Stakeholder Participation") OR (MH "Consumer Participation")) OR (TI(("service-user*" OR "service-recipient*" OR "patient*" or "consumer*" OR "client*" OR "caregiver*" OR "carer*" OR "famil*") N2 ("involvement" OR "participation" OR "engagement" OR "collaboration" OR "partnership*")) OR AB(("service-user*" OR "service-recipient*" OR "patient*" or "consumer*" OR "client*" OR "caregiver*" OR "carer*" OR "famil*") N2 ("involvement" OR "participation" OR "engagement" OR "collaboration*" OR "partnership*")))) AND (((MH "Education, Interdisciplinary") OR (MH "Interprofessional Relations+") OR (MH "Multidisciplinary Care Team+")) OR (TI("interprofession*" OR "inter-profession*" OR "interdisciplin*" OR "inter-disciplin*") OR AB("interprofession*" OR "inter-profession*" OR "interdisciplin*" OR "inter-disciplin*")))
Cochrane	(([mh "community participation"] OR [mh "stakeholder participation"]) OR (((service-user* OR service-recipient* OR patient* OR consumer* OR client* OR caregiver* OR carer* OR famil*) NEAR/2 (involvement OR participation OR engagement OR collaboration* OR partnership*)):ti,ab,kw)) AND (([mh "interprofessional education"] OR [mh ^"patient care team"] OR [mh "interprofessional relations"]) OR ((interprofession* OR inter-profession* OR interdisciplin* OR inter-disciplin*):ti,ab,kw))
Embase	(('patient participation'/exp OR 'patient engagement'/exp OR 'family participation'/exp OR 'family engagement'/exp) OR ((('service-user*' OR 'service-recipient*' OR 'patient*' or 'consumer*' OR 'client*' OR 'caregiver*' OR 'carer*' OR 'famil*') NEAR/2 ('involvement' OR 'participation' OR 'engagement' OR 'collaboration*' OR 'partnership*')):ti,ab,kw)) AND (('interprofessional education'/exp OR 'interprofessional learning'/exp OR 'interprofessional'/exp OR 'interdisciplinary education'/exp OR 'interdisciplinary communication'/exp OR 'interdisciplinary care'/exp OR 'interdisciplinary team'/exp OR 'interprofessional collaboration'/exp) OR (('interprofession*' OR 'interdisciplin*' OR 'inter-profession*' OR 'inter-disciplin*'):ti,ab,kw))

MEDLINE and PubMed Central via OVID	((Exp Community Participation/ OR Exp Stakeholder Participation/) OR ((("service-user*" OR "service-recipient*" OR "patient*" or "consumer*" OR "client*" OR "caregiver*" OR "carer*" OR "famil*") ADJ2 ("involvement" OR "participation" OR "engagement" OR "collaboration" OR "partnership*")).ti,ab,kf.)) AND ((exp Interprofessional Education/ OR Patient Care Team/ OR exp Interprofessional Relations/) OR (("interprofession*" OR "inter-profession*" OR "interdisciplin*" OR "inter-disciplin*").ti,ab,kf.))

Supplementary Table S3
Data extraction template

Title	
Authors	
Year of publication	
Country of origin	
Setting (research, practice, policy, etc.)	
Description of the setting	
Study design	
Participants	
Characteristics of service users	
Professional involved	
IPECP initiative (program, service)	
Extent of user involvement in interprofessional work	
Enablers	
Barriers	
Remarks	

Supplementary Table S4

McMaster critical appraisal for included studies -Qualitative

	Study Purpose	Literature	Sampling			Data collection					Data Analysis										
	**Q1**	**Q2**	**Q4a**	**Q4b**	**Q4c**	**Q5a**	**Q5b**	**Q5c**	**Q5d**	**Q5e**	**Q6a**	**Q6b**	**Q6c**	**Q6d**	**Q6e**	**Q6f**	**Q6g**	**Q6h**	**Q6i**	**Q6j**	**Q6k**
Bolin, 2014 [43]	Y	Y	Y	Y	NAD	Y	Y	N	N	Y	N	Y	Y	Y	Y	N	N	Y	Y	Y	Y
Carr et al. 2012 [39]	Y	Y	Y	NAD	NAD	Y	N	Y	NAD	NAD	NAD	Y	NAD	NAD	Y	NAD	NAD	NAD	NAD	Y	Y
Metersky et al., 2021 [31]	Y	Y	Y	Y	Y	Y	Y	N	Y	Y	Y	Y	NAD	Y	Y	Y	N	Y	N	Y	Y
Molenaar et al., 2018 [36]	Y	Y	Y	Y	Y	Y	Y	N	N	Y	Y	Y	Y	Y	Y	Y	Y	Y	Y	Y	Y
Philips et al., 2015 [41]	Y	Y	Y	Y	Y	N	Y	N	N	Y	NAD	Y	Y	Y	Y	Y	N	Y	Y	Y	Y
Reeves et al., 2015 [46]	Y	Y	Y	Y	Y	Y	Y	Y	N	Y	N	Y	NAD	Y	Y	Y	N	Y	Y	Y	Y
Sæbjørnsen & Willumsen, 2015 [42]	Y	Y	Y	Y	Y	Y	Y	Y	N	Y	Y	Y	Y	Y	Y	Y	N	Y	Y	Y	Y
Schoeb et al., 2019 [44]	Y	Y	Y	Y	Y	Y	Y	N	N	Y	Y	Y	NAD	Y	Y	Y	Y	Y	Y	Y	Y
Sitzia et al., 2006 [40]	Y	Y	Y	Y	Y	Y	Y	N	N	Y	Y	Y	NAD	Y	Y	Y	N	Y	Y	Y	Y
Tjia et al., 2021 [45]	Y	Y	Y	Y	Y	N	Y	N	N	NAD	Y	Y	NAD	Y	Y	Y	N	Y	Y	Y	Y
Valaitis,et al, 2019 [32]	Y	Y	Y	Y	Y	Y	Y	N	N	Y	Y	Y	Y	Y	Y	Y	Y	Y	Y	Y	Y

van Dongen et al. 2017 [35]	Y	Y	Y	NAD	Y	Y	Y	Y	NAD	Y	NAD	Y	Y	Y	Y	Y	Y	Y	Y	Y	Y
van Dongen et al. 2017 [36]	Y	Y	Y	Y	Y	Y	Y	Y	N	Y	N	Y	Y	Y	Y	Y	N	Y	Y	Y	Y
Worswick, 2015 [33]	Y	Y	NAD	NAD	NAD	NAD	NAD	NAD	NAD	NAD	NAD	Y	NAD	NAD	Y	NAD	NAD	NAD	NAD	Y	Y

Keys: Y= Yes, N = No, NAD = Not Addressed, N/A = Not Applicable

Questions:

Study Purpose: 1. Was the purpose and/or research question stated clearly? Literature: 2. Was relevant background literature reviewed? Study Design: 3a. What was the design? 3b. Was a theoretical perspective identified? 3c. Method(s) used Sampling: 4a. Was the process of purposeful selection described? 4b. Was sampling done until redundancy in data was reached? 4c. Was informed consent reached? Data Collection:

Descriptive Clarity 5a. Clear & complete description of site 5b. Clear & complete description of participants 5c. Role of researcher & relationship with participants 5d. identification of assumptions and biases of researcher Procedural rigour 5e. Procedural rigour was used in data collection strategies? Data Analyses: Analytical Rigour 6a. Data analyses were inductive? 6b. Findings were consistent with & reflective of data? Auditability 6c. Decision trail developed? 6d. Process of analyzing the data was described adequately? Theoretical Connections 6e. Did a meaningful picture of the phenomenon under study emerge? Overall Rigour - Was there evidence of the four components of trustworthiness? 6f. Credibility? 6g. Transferability? 6h. Dependability? 6i. Confirmability? Conclusions and Implications 6j. Conclusions were appropriate given the study findings? 6k. The findings contributed to theory development & future OT practice/ research?

Supplementary Table S5

McMaster critical appraisal for included studies -Quantitative

	Study Purpose	Literature	Study Design	Sampling		Outcomes		Intervention			Results			Drop-outs	Conclusions & Implications
	**Q1**	**Q2**	**Q3**	**Q4a**	**Q4b**	**Q5a**	**Q5b**	**Q6a**	**Q6b**	**Q6c**	**Q7a**	**Q7b**	**Q7c**	**Q8**	**Q9**
Koerner et al. 2014 [38]	Y	Y	RCT	Y	N	Y	Y	Y	NAD	NAD	Y	Y	Y	Y	Y

Keys: Y= Yes, N = No, NAD = Not Addressed, N/A = Not Applicable

Questions:

Study Purpose: 1. Was the purpose stated clearly? Literature: 2. Was relevant background literature reviewed? Design: 3 Was the design appropriate for the study question? Sample: 4a. Was the sample described in detail? 4b. Was sample size justified? Outcomes: 5a. Were the outcome measures reliable? 5b Were the outcome measures valid? Intervention: 6a. Intervention was described in detail? 6b. Contamination was avoided? 6c. Cointervention was avoided? Results: 7a. Results were reported in terms of statistical significance? 7b. Were the analysis method(s) appropriate? 7c. Clinical importance was reported? Drop-outs: 8. Drop-outs were reported? Conclusions and Implications: 9. Conclusions were appropriate given study methods and results

Supplementary Table S6

Mixed Methods Appraisal Checklist for Included Studies

	Screening		Qualitative					Quantitative Randomized Controlled Trials					Quantitative Non-Randomized					Quantitative Descriptive				Mixed Methods					
	**S1**	**S2**	**1.1**	**1.2**	**1.3**	**1.4**	**1.5**	**2.1**	**2.2**	**2.3**	**2.4**	**2.5**	**3.1**	**3.2**	**3.3**	**3.4**	**3.5**	**4.1**	**4.2**	**4.3**	**4.4**	**4.5**	**5.1**	**5.2**	**5.3**	**5.4**	**5.5**
Körner, 2013 [37]*	Y	Y	Y	Y	Y	Y	Y	-	-	-	-	-	-	-	-	-	-	Y	CT	Y	CT	Y	Y	Y	Y	Y	CT*

*no quality measures (trustworthiness, rigor, etc.) for qualitative data was mentioned

Keys: Y= Yes, N = No, CT = Can't tell, - = Not Applicable

Questions:

<u>Screening</u>: S1. Are there clear research questions? S2. Do the collected data allow to address the research questions? <u>Qualitative</u>: 1.1. Is the qualitative approach appropriate to answer the research question? 1.2. Are the qualitative data collection methods adequate to address the research question? 1.3. Are the findings adequately derived from the data?1.4. Is the interpretation of results sufficiently substantiated by data? 1.5. Is there coherence between qualitative data sources, collection, analysis and interpretation? <u>Quantitative randomized controlled trials</u>: 2.1. Is randomization appropriately performed? 2.2. Are the groups comparable at baseline? 2.3. Are there complete outcome data? 2.4. Are outcome assessors blinded to the intervention provided? 2.5 Did the participants adhere to the assigned intervention? <u>Quantitative non-randomized</u>: 3.1. Are the participants representative of the target population? 3.2. Are measurements appropriate regarding both the outcome and intervention (or exposure)? 3.3. Are there complete outcome data? 3.4. Are the confounders accounted for in the design and analysis? 3.5. During the study period, is the intervention administered (or exposure occurred) as intended? <u>Quantitative descriptive</u>: 4.1. Is the sampling strategy relevant to address the research question? 4.2. Is the sample representative of the target population? 4.3. Are the measurements appropriate? 4.4. Is the risk of nonresponse bias low? 4.5. Is the statistical analysis appropriate to answer the research question? <u>Mixed methods</u>: 5.1. Is there an adequate rationale for using a mixed methods design to address the research question? 5.2. Are the different components of the study effectively integrated to answer the research question? 5.3. Are the outputs of the integration of qualitative and quantitative components adequately interpreted? 5.4. Are divergences and inconsistencies between quantitative and qualitative results adequately addressed? 5.5. Do the different components of the study adhere to the quality criteria of each tradition of the methods involved?
